# Supplementary material for: Complexome profiling of the Chlamydomonas psb28 mutant reveals TEF5 as an early PSII assembly factor
Source: Plant Cell. 2025 Mar 17;37(6):koaf055. doi: 10.1093/plcell/koaf055 (PMC12164590; doi:10.1093/plcell/koaf055)
Supplement: koaf055_Supplementary_Data [file koaf055_supplementary_data.zip › psb28_interactive.zip!/plots/intensity/qualityPlots/peptides/TEF24_Cre09.g394850.t1.2.html]

### Cre09.g394850.t1.2

Trivial name: TEF24  
SeqCoverage [median]: 9.50  
Mass: 49.91 kDa  
  
 

| Sequence | MissedCleavage | Mass [Da] | wtIntensity | muIntensity | Score |
| --- | --- | --- | --- | --- | --- |
| ALALNPDLAR | 0 | 1052.60 | 24963.45 | 56654.66 | 148.52 |
| GLAAAGTAGGLGTASLTSK | 0 | 1602.86 | 11101.51 | 20638.44 | 198.23 |
| LVTGLASLYALDMAIKK | 1 | 1806.03 | 2615.38 | 0.00 | 60.47 |
|  |  |  | 38680.3 | 77293.1 |  |
